# SPERM FACTORS AND EGG ACTIVATION: Phospholipase C zeta (PLCZ1) and the clinical diagnosis of oocyte activation deficiency

**DOI:** 10.1530/REP-21-0458

**Published:** 2022-03-21

**Authors:** C Jones, X Meng, K Coward

**Affiliations:** 1 Nuffield Department of Women’s & Reproductive Health, University of Oxford, Women’s Centre, John Radcliffe Hospital, Headington, Oxford, UK

## Abstract

Oocyte activation deficiency (OAD) remains the predominant cause of total/low fertilization rate in assisted reproductive technology. Phospholipase C zeta (PLCZ1) is the dominant sperm-specific factor responsible for triggering oocyte activation in mammals. OAD has been linked to numerous PLCZ1 abnormalities in patients experiencing failed *in vitro* fertilization or intracytoplasmic sperm injection cycles. While significant efforts have enhanced our understanding of the clinical relevance of PLCZ1, and the potential effects of genetic variants upon functionality, our ability to apply PLCZ1 in a diagnostic or therapeutic role remains limited. Artificial oocyte activation is the only option for patients experiencing OAD but lacks a reliable diagnostic approach. Immunofluorescence analysis has revealed that the levels and localization patterns of PLCZ1 within sperm can help us to indirectly diagnose a patient’s ability to induce oocyte activation. Screening of the gene encoding PLCZ1 protein is also critical if we are to fully determine the extent to which genetic factors might play a role in the aberrant expression and/or localization patterns observed in infertile patients. Collectively, these findings highlight the clinical potential of PLCZ1, both as a prognostic indicator of OAD and eventually as a therapeutic agent. In this review, we focus on our understanding of the association between OAD and PLCZ1 by discussing the localization and expression of this key protein in human sperm, the potential genetic causes of OAD, and the diagnostic tools that are currently available to us to identify PLCZ1 deficiency and select patients that would benefit from targeted therapy.

## Male infertility

The incidence of infertility is rising as the global population continues to grow and is now considered to affect 8–12% of couples worldwide (Agarwal *et al.* 2021). According to the European Society for Human Reproduction and Embryology, the causes of infertility can be classified as sole-male factor (20–30%), sole-female factor (20–35%), due to both partners (25–40%), or idiopathic (10–20%) (ESHRE 2018). The Human Fertilization and Embryology Authority (HFEA) reports that the most common reasons for patients seeking *in vitro* fertilization (IVF) treatment are male infertility (37%), female infertility (31%), and cases involving unexplained infertility (32%) (HFEA 2018). Currently, male infertility is responsible for 25–30% of all cases of infertility and can arise as a result of testicular deficiency (such as varicocele), post-testicular impairment, or other genetic/molecular abnormalities that affect the production, quality, and function of sperm (Esteves 2016, Leslie *et al.* 2020, Agarwal *et al.* 2021). The field of assisted reproductive technology (ART) is revolutionizing the field of reproductive medicine and strives continuously to provide infertile patients with access to appropriate and efficient treatments. Indeed, intracytoplasmic sperm injection (ICSI) has now become the most commonly applied technique and accounts for two-thirds of all global ART treatments; conventional IVF only accounts for one-third of all treatments (Haddad *et al.* 2021). Regardless of the undoubtable success of ART, total fertilization failure (TFF), in which all of the mature oocytes retrieved from the female fail to fertilize in one cycle, occurs in 5–20% of all IVF cycles and 1–3% of all ICSI cycles (Cardona Barberán *et al.* 2020).

The principal cause of TFF following ICSI is oocyte activation deficiency (OAD). This is when an oocyte fails to undergo further maturation and complete fertilization following sperm fusion. Over recent years, OAD has been linked to numerous PLCZ1 abnormalities in patients experiencing failed IVF or ICSI cycles (Nikiforaki *et al.* 2016, Yeste *et al.* 2016*a*). Numerous studies have shown that PLCZ1 is responsible for inducing Ca^2+^ oscillations in the activating oocyte; these oscillations control crucial cell signalling pathways in the early embryo. Since the discovery of PLCZ1 in 2002, a significant body of research has demonstrated the fundamental role of PLCZ1 as the sperm-specific oocyte activation factor and have provided strong evidence to support a causal link between PLCZ1 deficiency and male infertility (Saunders *et al.* 2002, Sanders & Swann 2016, Swann & Lai 2016, Parrington *et al.* 2019). Previous research has demonstrated that patients with reduced levels of PLCZ1, or with a total absence of PLCZ1, are unable to initiate the Ca^2+^ oscillations that are required for successful oocyte activation. Furthermore, some reports have shown that levels of PLCZ1 protein and mutations in the gene encoding PLCZ1, are strongly associated with male infertility/subfertility (Kashir 2020). In addition, reports have shown that sperm from fertile patients can evoke variable Ca^2+^ responses; thus, such sperm may be responsible for the failure of some cycles (Swann 2018, Meng *et al.* 2020). Further investigations successfully demonstrated the association between the localization and expression levels of PLCZ1 and successful ICSI cycles (Yelumalai *et al.* 2015).

Yoon *et al.* were the first to inject human sperm from patients with a history of ICSI failure into mouse oocytes; this practice identified the absence of the normal Ca^2+^ oscillations and resulted in oocyte activation failure (Yoon *et al.* 2008). In addition, these authors demonstrated that the co-injection of *Plcz1* mRNA into mouse oocytes, alongside PLCZ1-deficient sperm, successfully rescued OAD, thus reinforcing the fundamental role of PLCZ1 for the treatment of oocyte activation (Yoon *et al.* 2008). These findings highlighted the fact that investigating the specific expression of PLCZ1 protein in human sperm can facilitate our understanding of their ability to trigger oocyte activation. Ever since, research has become increasingly focused on the design of therapeutic and diagnostic applications for PLCZ1 and the translation of such technology to the clinical setting (Kashir *et al.* 2013, Nomikos *et al.* 2013*a*, *b*). In this review, we focus on our understanding of the association between OAD and PLCZ1 by discussing the localization and expression of this key protein in human sperm, the potential genetic causes of OAD, and the current diagnostic tools that are available to us to identify cases of PLCZ1 deficiency.

## What is known about the localization patterns and expression levels of PLCZ1 in sperm

**Figure 1:**
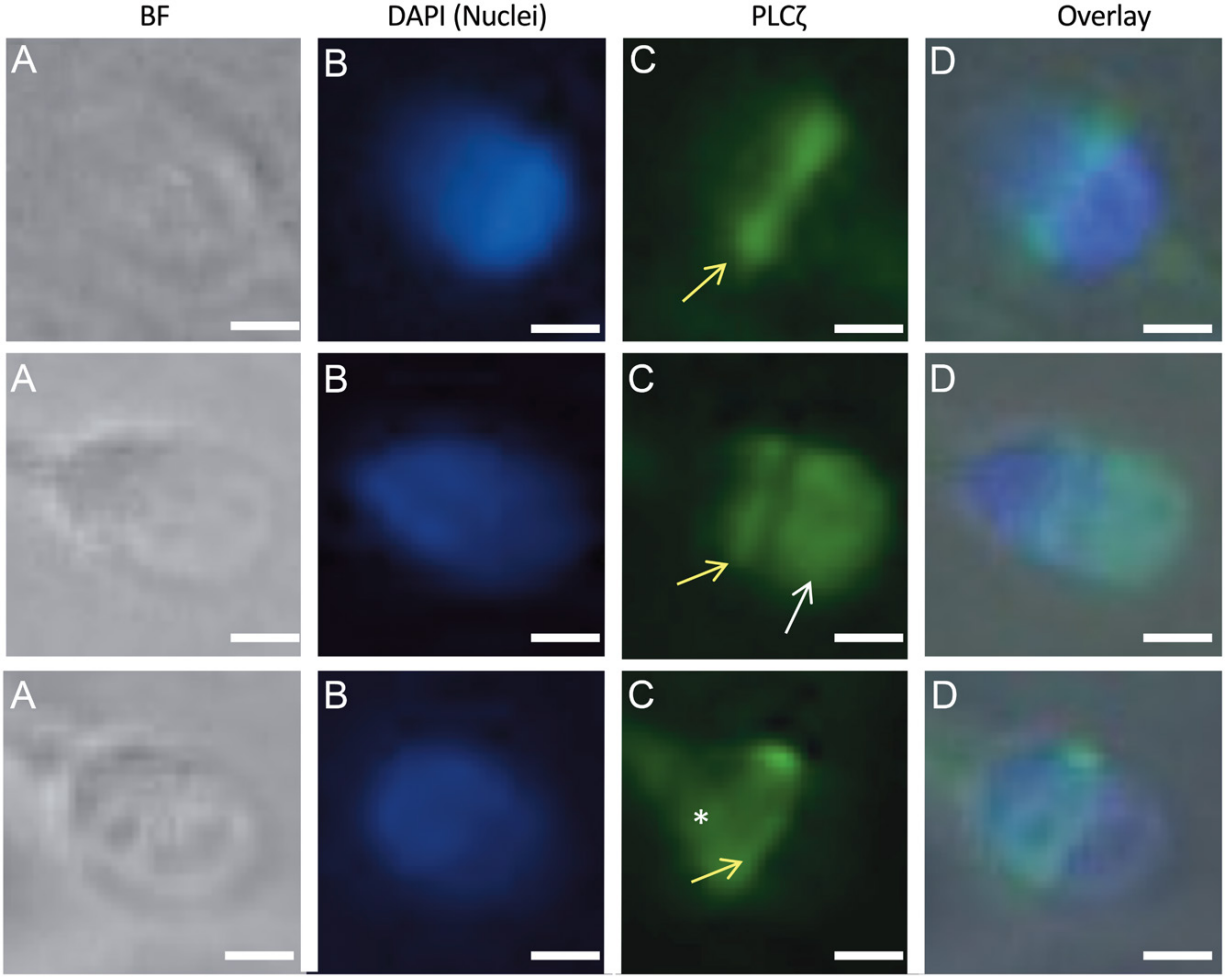
Representative immunofluorescence images of PLCZ1 localization in sperm from a fertile male. Images were captured as (A) bright-field or stained with (B) DAPI and (C) FITC-PLCZ1. Merged images are shown in (D). PLCZ1 is expressed in the equatorial (yellow arrows), acrosomal (white arrow), and postacrosomal (asterisk) regions in the sperm. Scale bar = 5 μm. Reproduced with permission from Meng *et al.* (2020).

Previous research has shown that the specific localization patterns and expression levels of PLCZ1 in sperm are associated with OAD. Immunofluorescence analysis has revealed that PLCZ1 is predominantly localized in the equatorial segment of human sperm but is also localized in the acrosome, post-acrosome, tail, or a combination of these locations (Fig. 1) (Grasa *et al.* 2008, Kashir *et al.* 2013, Yelumalai *et al.* 2015, Ferrer-Vaquer *et al.* 2016, Yeste *et al.* 2016*b*). In human sperm, the most dominant region for PLCZ1 localization is the equatorial region; this makes biological sense because this would facilitate the release of PLCZ1 protein into the ooplasm following gamete fusion to initiate the sequence of events that are vital for embryogenesis (Nomikos *et al.* 2012). The localization of PLCZ1 in multiple spatial regions has raised speculation that different populations of this protein may potentially be responsible for differential functions (Grasa *et al.* 2008), although this theory has yet to be proven. For example, Grasa *et al.* reported that the equatorial localization of PLCZ1 remained consistent during sperm capacitation and the acrosome reaction and suggested that PLCZ1 localization in the acrosomal region of the sperm head may mediate the acrosome reaction rather than triggering oocyte activation (Grasa *et al.* 2008). It is possible that the acrosomal population of PLCZ1 may play a role in fertilization; however, due to the timing of the acrosome reaction in relation to sperm–oocyte fusion, there are significant doubts with regards to what functional role this particular population of PLCZ1 might play. Furthermore, reports have demonstrated a potential shift in PLCZ1 localization to the post-acrosomal region following capacitation (Grasa *et al.* 2008, Young *et al.* 2009). Interestingly, another study put forward the hypothesis that a truncated isoform of PLCZ1, inexplicably termed ‘NYD-SP7’ was localized within the acrosomal regions of murine and human sperm and served as a decapacitation factor (Bi *et al.* 2009). However, confusion remains regarding the precise nature and intention of this study since NYD-SP7 lacked the inherent domain structure of the PLCZ1 protein, thus rendering it functionally inactive. Some researchers have reported the localization of PLCZ1 to the sperm tail. It remains unknown whether these findings are legitimate or merely represent a technical flaw related to immunological detection technology. Examination of the current literature clearly shows that various different PLCZ1 localization patterns have been reported in sperm, although most researchers seem to agree that the most valid and justifiable localization pattern of PLCZ1 is in the equatorial region. However, it is important that future studies identify the specific reasons for potential PLCZ1 localization in the sperm tail. A previous study by Bedford-Guaus *et al*. (2011) achieved calcium responses in oocytes by simply injecting the tails of equine sperm. Thus, we cannot omit the possibility that PLCZ1 localized in the tail may exert functionality in terms of activation or in downstream processes. However, Kashir *et al.* claimed that the localization of PLCZ1 in the sperm tail is simply an artefact; these authors noted that antibody specificity remains a notable problem and that we should disregard the localization of PLCZ1 in the sperm tail (Kashir *et al.* 2020). Researchers have yet to consider the potential function of PLCZ1 populations in the sperm tail; further research is needed to address this possibility, especially in human sperm (Bedford-Guaus *et al.* 2011).

The most demanding conundrum in PLCZ1 research is the significant variability of the expression levels and localization patterns of PLCZ1 that has been described in both fertile donors and infertile patients (Grasa *et al.* 2008, Kashir *et al.* 2013). Studies have reported significant variation in both the expression level and localization pattern of PLCZ1 within the same ejaculate and when compared between different donors and patients (Yoon *et al.* 2008, Heytens *et al.* 2009, Kashir *et al.* 2011*a*, *b*, 2012*a*, Escoffier *et al.* 2015*a*, Ferrer-Vaquer *et al.* 2016, Javadian-Elyaderani *et al.* 2016, Dai *et al.* 2020, Rahimizadeh *et al.* 2020). These variations in PLCZ1 content between the ejaculates of fertile and infertile men raise significant questions as to why such disparity occurs. One explanation could be that levels of PLCZ1 are reduced during spermatid elongation; however, further investigations are needed to specifically determine the localizations patterns of PLCZ1 within the testes, particularly in humans.

Previous immunofluorescent analysis of single sperm, from both fertile and infertile men, also showed wide variation in two key parameters of interest: total levels and localization patterns of PLCZ1 (Grasa *et al.* 2008, Kashir *et al.* 2013). However, despite the fact that some fertile controls can exhibit similar levels of PLCZ1 expression to infertile patients, immunofluorescence analyses have clearly confirmed that the relative fluorescence (RF) intensity of PLCZ1 in human sperm is significantly lower in OAD patients than in fertile males (Heytens *et al.* 2009, Kashir *et al.* 2013), thus providing us with the capability to perform diagnostic assays.

Other researchers have reported the inability of patients with globozoospermia, a condition in which the sperm are round and lack the acrosome, to evoke long-term Ca^2+^ oscillations following the injection of their sperm into mouse oocytes. Previous studies have shown that PLCZ1 is localized in the perinuclear theca of the equatorial and post-acrosomal region of human sperm (Escoffier *et al*. 2015*b*). Patients with globozoospermia have absent or abnormal levels of PLCZ1 in their sperm and show punctuated localization patterns (Heytens *et al.* 2009, Kashir *et al.* 2012*b*). This is not surprising as an individual with DPY19L2-dependent globozoospermia was found to produce defective sperm featuring loss of the acrosome and surrounding materials including PLCZ1 (Escoffier *et al.* 2015*b*). Subsequent studies showed that patients diagnosed with partial or complete globozoospermia are unable to fertilize oocytes naturally and that this deficiency is related to reduced levels or the total absence of PLCZ1 (Taylor *et al.* 2010, Abadi *et al.* 2016, Tavalaee & Nasr-Esfahani 2016, Tavalaee *et al.* 2018, Cheung *et al.* 2020).

**Figure 2:**
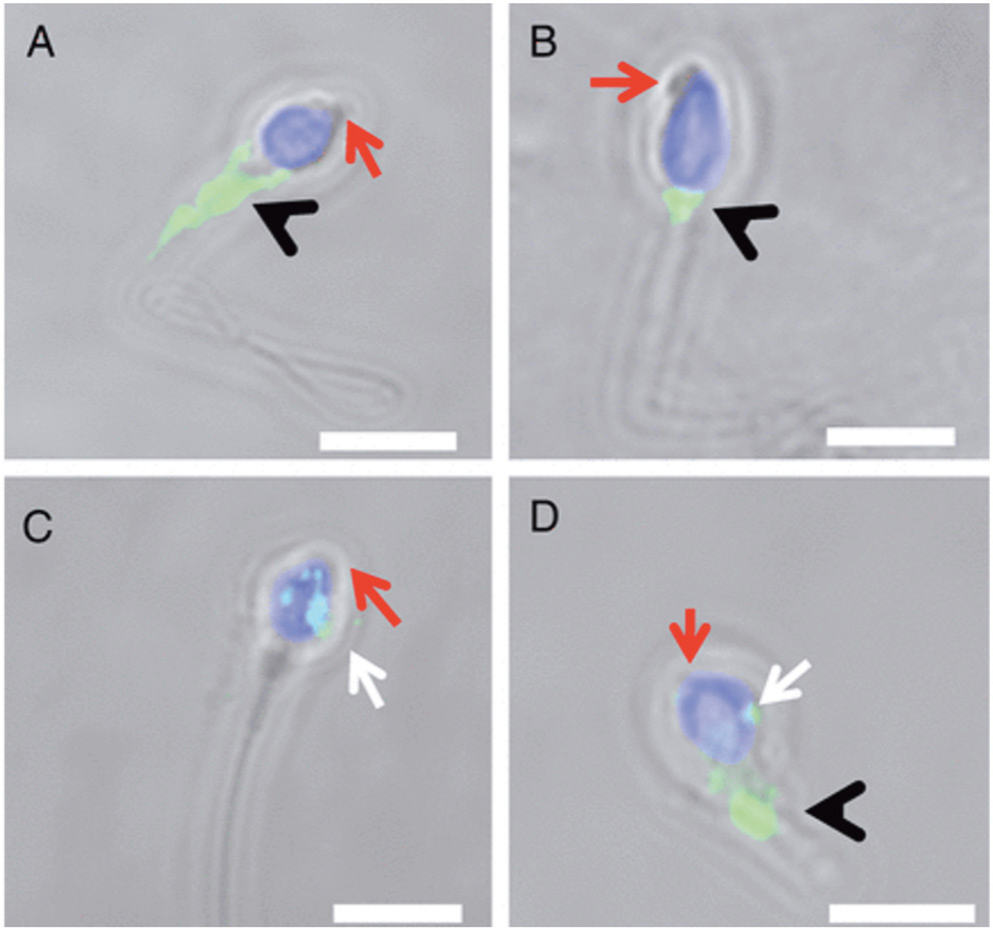
Representative confocal images of PLCZ1 immunofluorescence in motile sperm organelle morphology examination (MSOME)-selected globozoospermic sperm exhibiting an acrosomal bud (red arrow). PLCZ1 (pale green staining) was localized to the midpiece (black arrowheads, A and B), or as a punctate pattern in the sperm head (white arrows, C), or in combination (D). White scale bar represents 5 µm. Reproduced with permission from Kashir *et al*. (2012*b*).

Motile sperm organelle morphology examination (MSOME) has been used to study the specific morphology of globozoospermic sperm and successfully identify a sub-population of sperm exhibiting a small acrosomal bud (Sermondade *et al.* 2011). Subsequent ICSI cycles, involving the injection of globozoospermic sperm featuring these small acrosomal buds, permitted globozoospermic patients to achieve live birth without the use of artificial oocyte activators (AOAs) (Sermondade *et al.* 2011). Given these interesting findings, Kashir *et al.* identified that almost half (43%) of the sperm exhibiting acrosomal bud morphology expressed PLCZ1 within their head regions (Fig. 2) (Kashir *et al.* 2012*b*), albeit with highly abnormal localization patterns. Thus, the use of MSOME to identify acrosomal buds in the sperm heads of globozoospemic patients can provide a useful tool with which to guide clinical treatment by allowing the selection of sperm containing PLCZ1 from populations of sperm that do not contain PLCZ1.

The procedure used for semen analysis comprises basic macroscopic and microscopic diagnostic tests, including volume, concentration, motility, and viability; these tests are performed by trained clinical scientists in ART laboratories. However, tests relating to the functional aspects of the sperm, such as DNA fragmentation, mitochondrial function, and reactive oxygen species, require the use of more advanced techniques which are often not available in ART units (Agarwal *et al.* 2021). At present, the specific causes of male factor infertility or subfertility remain poorly understood (Agarwal *et al.* 2021). Furthermore, human sperm are far from being homogeneous and only a small proportion of sperm are considered as being ‘normal’ in a given semen sample. In ART laboratories, clinical scientists select, when possible, the best sperm to use for ICSI; however, the selection process is still very subjective and, in some cases, normozoopermic sperm are not available. We know that PLCZ1 is expressed at the early stage of spermatogenesis in the testis; thus, events that damage or alter spermatogenesis may contribute to variations of PLCZ1 expression in subsequent stages. With this in mind, studies have assessed the correlation between PLCZ1 expression and localization and sperm integrity. Researchers have reported different conclusions in terms of the correlation between PLCZ1 and DNA oxidation status. One group reported that higher levels of oxidative stress were correlated with lower PLCZ1 expression levels; however, other groups found no evidence of such a correlation (Park *et al.* 2015, Janghorban-Laricheh *et al.* 2016, Tavalaee *et al.* 2017). Some authors argued that this discrepancy may be due to the assays used. Furthermore, low semen quality, such as in patients with varicocele (a chronic genital heat stress condition), has been shown to have low levels of PLCZ1. It has been shown that PLCZ1 is heat-sensitive; therefore, this condition may disrupt the final translation of the protein (Sanusi *et al.* 2015). Accordingly, sperm from infertile males with teratozoospermia (abnormal head morphology) have previously been demonstrated to exhibit abnormal localization or the absence of PLCZ1 but showed a variance in PLCZ1 expression in comparison to controls (Azad *et al.* 2018). However, in another report, involving patients with asthenoteratozoospermia, in which motility and morphology are abnormal, showed no difference in PLCZ1 localization (Rahimizadeh *et al.* 2020). Other authors reported a correlation between sperm health and PLCZ1; however, the authors were not able to identify a specific correlation between morphology and PLCZ1 (Kashir *et al.* 2020). Additional studies, with larger cohorts, are now needed to investigate PLCZ1 with respect to abnormal and normal sperm morphology. We know that in the case of TFF, sperm are unable to induce Ca^2+^ oscillations. Furthermore, studies have shown that within an ejaculate, human sperm are able to cause different patterns of Ca^2+^ spikes in oocytes, thus reinforcing the idea that each sperm may contain a different amount of PLCZ1 or that the level of PLCZ1 activity in each sperm is variable (Ferrer-Buitrago *et al.* 2018). In addition, we know that PLCZ1 activity differs across species; studies have shown that a certain amount of PLCζ is required for the oocyte to achieve successful embryo development and that this amount varies across different species (Yoon *et al.* 2008, Nomikos *et al.* 2013*b*). This observation concurs with other studies reporting variability in the levels and localization patterns of PLCZ1 in fertile and infertile men. This raises the question as to why sperm have different levels, localization patterns, and functional activities.

Most notably, PLCZ1 levels, localization patterns, and the proportion of human sperm exhibiting PLCZ1, have been found to correlate significantly with fertilization rates following ICSI, thus indicating that PLCZ1 is a promising biomarker for diagnosing OAD and predicting the outcome of ICSI (Yelumalai *et al.* 2015, Nazarian *et al.* 2019). However, this concept has been questioned since another study concluded that PLCZ1 levels and localization patterns were not correlated with ICSI outcome (Ferrer-Vaquer *et al.* 2016). Similar to findings in humans, a recent study, conducted in stallions, reported that the injection of sperm with higher PLCZ1 protein levels into bovine oocytes led to a better embryonic cleavage rate (Amoroso-Sanches *et al.* 2019). Collectively, the existing body of evidence clearly indicates that PLCZ1 is a practical clinical biomarker that can be used to indirectly predict the oocyte activation ability of a given sperm sample. However, immunocytochemical staining alone is not sufficient to fully determine the PLCZ1 status of sperm. Screening of the gene encoding the PLCZ1 protein is also critical if we are able to fully determine the extent to which genetic factors might play a role in the aberrant expression and/or localization patterns observed in infertile patients.

## *PLCZ1* gene abrogation, recurrent inheritance mutations, and protein dysfunction

The human *PLCZ1* gene consists of 15 exons and is located on the reverse strand of chromosome 12. This gene encodes a PLCZ1 protein that is composed of a series of key functional domains (EF, X, Y, and C2) with a linker region that connects the catalytic X and Y domains. An obvious hypothesis is that PLCZ1 deficiency is linked to genetic alterations in the *PLCZ1* gene or the molecular mechanisms that govern the expression of this gene. Such alterations could readily manifest in a dysfunctional protein and exert a direct impact on oocyte activation capability. Consequently, significant efforts have been made to study PLCZ1 at the genomic level. Critically, several mutations have been detected in the human *PLCZ1* gene; some of these mutations have also been demonstrated to impair the functionality of the translated protein (Table 1) (Heytens *et al.* 2009, Kashir *et al.* 2011*c*, Escoffier *et al.* 2015*a*, Ferrer-Vaquer *et al.* 2016, Torra-Massana *et al.* 2019, Kashir 2020). Several of these mutations have been modelled in the laboratory to confirm whether the functionality of the translated protein was impaired under normal physiological conditions.

**Table 1:** Literature-based summary of PLCZ1 mutations identified in infertile males with/without follow-up functional investigation. Reproduced from Kashir (2020) with permission.

Mutation	Domain affected	*In vitro* phenotype	*In vivo* phenotype	References
I120M	EF-X linker	Predicted alteration of local protein folding	OAD; low fertilization success	Torra-Massana *et al*. (2019)
C196X	X	Predicted alteration of local protein folding	OAD; reduced/absent PLCZ1 in patient sperm; abnormal PLCZ1 localization	Dai *et al*. (2020)
C196*	X	No recombinant protein produced by mammalian cells; reduced activation success following cRNA injection in mouse oocytes	OAD; reduced/absent PLCZ1 in patient sperm; low fertilization success	Mu *et al*. (2020), Yan *et al.* (2020)
R197H	X	Predicted alteration of local protein folding	OAD; low fertilization success	Ferrer-Vaquer *et al.* (2016), Torra-Massana *et al.* (2019)
L224P	X	Predicted alteration of local protein folding	OAD; low fertilization success	Torra-Massana *et al.* (2019)
H233L	X	Reduced expression in mammalian cells; reduced/absent oscillations following cRNA injections in mouse oocytes; reduced embryogenesis in mouse; predicted alteration of local protein folding	OAD; reduced/absent PLCZ1 in patient sperm; abnormal PLCZ1 localization; low fertilization success	Kashir *et al*. (2011*b*,*b*, 2012*a*), Ferrer-Vaquer *et al.* (2016), Torra-Massana *et al.* (2019)
L246F	X	Predicted alteration of local protein folding	OAD; reduced/absent PLCZ1 in patient sperm; abnormal PLCZ1 localization	Dai *et al.* (2020)
L277P	X	Predicted alteration of local protein fold; reduced activation success following cRNA injection in human oocytes	OAD; reduced/absent PLCZ1 in patient sperm; low fertilization success	Yan *et al.* (2020)
T324fs	X-Y linker	Truncated recombinant protein produced by mammalian cells; reduced activation success following cRNA injection in mouse oocytes	OAD; low fertilization success	Mu *et al.* (2020)
V326K fs*25	X-Y linker	Predicted frameshift truncation of protein	OAD; low fertilization success	Torra-Massana *et al.* (2019)
S350P	Y	Predicted alteration of local protein folding	OAD; reduced/absent PLCZ1 in patient sperm; abnormal PLCZ1 localization	Dai *et al*. (2020)
N377del	Y	Predicted alteration of local protein fold; no activation success following cRNA injection in human oocytes	OAD; reduced/absent PLCZ1 in patient sperm; low fertilization success	Yan *et al*. (2020)
A384V	Y	Predicted alteration of local protein fold; n +C16o activation success following cRNA injection in human oocytes	OAD; reduced/absent PLCZ1 in patient sperm; low fertilization success	Yan *et al.* (2020)
H398P	Y	Reduced expression in mammalian cells; reduced/absent oscillations following cRNA injections in mouse oocytes; predicted alteration of local protein folding	OAD; reduced/absent PLCZ1 in patient sperm; abnormal PLCZ1 localization	Heytens *et al*. (2009), Kashir *et* *al.* (2011*b*,*b*, 2012*a*)
R412fs	Y	Truncated recombinant protein produced by mammalian cells; reduced activation success following cRNA injection in mouse oocytes	OAD; low fertilization success	Mu *et al.* (2020)
P420L	Y	Reduced recombinant protein produced by mammalian cells; reduced activation success following cRNA injection in mouse oocytes	OAD; low fertilization success	Mu *et al.* (2020), Yuan *et al.* (2020*b*)
K448N	Y	Predicted alteration of local protein fold; reduced activation success following cRNA injection in human oocytes	OAD; reduced/absent PLCZ1 in patient sperm; low fertilization success	Yan *et al*. (2020)
I489F	C2	Reduced/absent oscillations following cRNA injections in mouse oocytes; reduced embryogenesis in mouse; predicted alteration of local protein fold; similar enzymatic properties but dramatically reduced substrate binding	OAD; reduced/absent PLCZ1 in patient sperm; abnormal PLCZ1 localization	Escoffier *et al.* (2015*a*), Nomikos *et al*. (2017)
S500L	C2	Predicted alteration of local protein folding	OAD; low fertilization success	Ferrer-Vaquer *et al.* (2016), Torra-Massana *et al*. (2019)
R553P	C2	Reduced/absent fertilization following cRNA injections in mouse oocytes; predicted alteration of local protein fold; mouse fertilization and embryogenesis comparable following injection of higher levels of mutant cRNA	Comparable levels of PLCZ1 in patient sperm	Yuan *et al.* (2020*a*)
L576P	C2	Predicted to affect C2 domain structure or C2-catalytic domain interaction	Low fertilization success; live-birth without AOA in the third cycle	Yuan *et al.* (2020*b*)
M578T	After C2	Predicted alteration of local protein fold; No activation success following cRNA injection in human oocytes	OAD; reduced/absent PLCZ1 in patient sperm; low fertilization success	Yan *et al.* (2020), Yuan *et al.* (2020*b*)

AOA, artificial oocyte activation; OAD, oocyte activation deficiency; PLCZ1, phospholipase C zeta.

Heytens *et al.* were the first to identify a mutation in the *PLCZ1* gene. These authors screened the DNA of a normozoospermic male with OAD and identified a substitution mutation (H398P) that resulted in a single amino acid change in the final protein; this mutation exerted adverse effects upon the functional capability of the protein (Heytens *et al.* 2009). At this time, the authors used three-dimensional (3D) structural modelling to show that the substitution mutation destabilized the protein structure and interfered with its ability to bind to substrate. Further investigation demonstrated that this mutation abolished the hydrolytic activity of the PLCZ1 protein and a general failure to elicit Ca^2+^ oscillations in the ooplasm (Nomikos *et al.* 2011). Subsequently, a second mutation (H233L) was discovered in the same patient. Additional inheritance analysis showed that the H398P and H233L mutations were located on different alleles and that one mutation (H233L) was inherited *via* the maternal lineage (Kashir *et al.* 2011*c*). Subsequent genotyping of single sperm from this infertile patient revealed that all of his sperm exhibited either the H398P or H233L mutation but never both, thus confirming that the two mutations were heterozygous. These findings indicated, for the first time, that *PLCZ1*-linked infertility can be inherited *via* the maternal lineage (Kashir *et al.* 2011*c*). In addition, the transfection of both mutations into human embryonic kidney cells (HEK293T) *via* plasmid DNA showed that the fluorescence level of recombinant mutant protein was significantly lower when a single mutation was transfected, thus indicating that a combination of these mutations may influence the stability of the protein more than a single mutation (Kashir *et al.* 2012*a*).

Subsequent research identified a homozygous mutation (I489F) in two brothers with normozoospermia who experienced TFF following ICSI and indicated that another brother (who was fertile) possessed the same mutation but in a heterozygous state. In addition, 3D modelling and liposome-binding assays showed that this mutation caused an amino acid substitution that led to a significant alteration in enzymic activity (Escoffier *et al.* 2015*a*, Nomikos *et al.* 2017).

Since then, two novel heterozygous mutations and a previously reported mutation have been identified in PLCZ1 (S500l, R197H, & H233L) in patients with failed fertilization; the researchers involved comments that heterozygote mutations may only reduce the functionality of the protein, as reported previously. However, no functional analyses were performed to investigate whether these mutations could specifically impair PLCZ1 functionality (Ferrer-Vaquer *et al.* 2016). More recently, Torra-Massana *et al.* reported not only the further detection of R197H, H233L, and S500L but also discovered three newly identified mutations (I120M, L224P, and V326K fs*25, a frameshift mutation) (Torra-Massana *et al.* 2019). Further bioinformatics analysis (involving Phyre2 software) suggested that the L224P and V326K fs*25 mutations exert detrimental effects on PLCζ protein function (Torra-Massana *et al.* 2019). These authors subsequently microinjected cRNA of their corresponding mutations into *in vitro*-matured human oocytes and found that the R197H and H233L mutations resulted in low activation rates (30 and 30.77%, respectively). In contrast, the L224P and S500L mutations had no influence on oocyte activation rates (64.29 and 60%, respectively). These findings highlighted the fact that not all genetic variations are detrimental to the functionality of PLCZ1 protein. Furthermore, the V326K fs*25 mutation was located in the XY-linker region, an important region that regulates enzyme activity; this mutation was caused by the deletion of two nucleotides (A and G), thus resulting in a frameshift and a premature stop codon downstream of the mutation. This was the first *PLCZ1* mutation to be identified in the XY-linker region. Bioinformatics analysis further indicated that the V326K fs*25 mutation would inevitably result in the translation of a truncated form of PLCZ1 protein. In line with these predictions, the injection of a truncated form of *PLCZ1* cRNA into human *invitro*-matured oocytes resulted in TFF (0/10), thus demonstrating that this particular mutation can exert a devastating effect on protein function (Torra-Massana *et al.* 2019).

More recently, Dai *et al.* identified a novel nonsense mutation in *PLCZ1* (C196X, the substitution of cysteine with an undetermined amino acid) and two novel missense mutations (S350Pand L246F) (Dai *et al.* 2020). These mutations were identified from three infertile males who experienced low fertilization rates (0–7.7%). Further 3D modelling suggested that the C196X mutation resulted in a premature termination codon that may impair protein function. In line with this, the infertile patient who exhibited the C196X mutation showed no PLCZ1 expression in his sperm. The other two mutations, S350P and L246F, also showed hydrogenic changes in the secondary structure of PLCZ1 protein. Interestingly, PLCZ1 was found to be expressed in sperm from patients exhibiting these two mutations. Unfortunately, the authors did not ascertain whether PLCZ1 expression levels were also reduced in these mutant sperm. Nevertheless, all three patients received AOA treatment in subsequent ICSI cycles and achieved significantly improved fertilization rates (from 4.4 to 56%), resulting in two successful ongoing pregnancies.

Research by Mu *et al*. further identified five mutations in four different families: C196*, R197H, P420L, R412fs, and T324fs). Interestingly, one mutation was recurrent in two families (C196*) and another (T324fs) was located within the XY linker. So far, only one other mutation has been identified in this critical part of the gene (Mu *et al.* 2020). Mu *et al*. compared the level of expression of each of these mutations *in vitro* and noted that all five mutations led to a significantly reduced level, or the total absence, of the final protein. Furthermore, reduced pronuclei formation was observed when the corresponding cRNAs were injected into mouse oocytes, thus suggesting that these mutations affected the functionality of PLCZ1 (Mu *et al.* 2020). All four families achieved successful fertilization after AOA treatment. Another study reported one known mutation and five novel mutations (C196*, M578T, A384V, N377del, L277P, and K448N); two families presented with the C196* mutation (Yan *et al.* 2020). Injection of cRNAs corresponding to the L277P and K448N mutations led to reduced pronuclear formation (27 and 11%, respectively) upon oocyte injection in comparison to the WT (86%). However, injection of cRNAs corresponding to M578T, A384V, and N377del failed to induce pronuclear formation. It is important to note that two of these mutations were homozygous, while the other three were heterozygous, thus re-affirming the autosomal recessive inheritance of PLCZ1. In this study, 14 patients were recruited by virtue of their low fertilization rate or due to TFF and the detection of sperm defects by the mouse activation assay (MOAT) (Yan *et al.* 2020). Only five patients were chosen for subsequent AOA treatment because of their mutations and the lack of protein PLCZ1 expression, as determined by Western blotting. Four patients opted for AOA treatment. Unfortunately, this study failed to report the expression levels and localization patterns of PLCZ1 for each patient (Yan *et al.* 2020). Also, genetic screening has assisted in the diagnosis and treatment of infertility detected in five cases, further investigations are now needed to elucidate the cause of infertility in the other nine patients (Yuan *et al.* 2020*a*). Furthermore, studies have shown that patients with heterozygous mutations presented with fertilization failure or were infertile. However, other reports have shown that the fathers of these patients were fertile; therefore, such inheritance may not necessarily affect fertility (Torra-Massana *et al.* 2019).

More recently, another mutation was discovered in a patient with normal semen parameters (R553P) (Yuan *et al.* 2020*a*). Western blotting analysis revealed the presence of PLCZ1 protein in the patient’s sperm; however, TFF was observed after ICSI in 50 oocytes. Further analysis revealed that the mutation was likely to disrupt protein folding and thus impact on protein functionality. The authors reported that the female patient had achieved a successful pregnancy with a previous partner, thus reinforcing the link between PLCZ1 deficiency and fertilization failure. The latest mutation that we would like to report in this review by Yuan *et al*. has extended our knowledge relating to failed fertilization and autosomal recessive inheritance patterns, particularly with regards to their discovery of three novel mutations in two different males (Yuan *et al.* 2020*b*). Indeed, in this study, DNA sequencing of one patient experiencing TFF showed that the patient had inherited the M578T mutation from his father and P420L from his mother (Yuan *et al.* 2020*b*). This study provided further evidence that PLCZ1 mutations can be inherited from the maternal lineage and that more attention is needed to focus on the inheritance of subfertility. The other patient with low fertilization capacity possessed the L576P mutation; unfortunately, no other genetic data were acquired for this particular patient. Interestingly, none of these mutations exerted impact on the transcriptional expression of PLCZ1 (Yuan *et al.* 2020*b*). The authors assumed that the homozygous L576P mutation may result in a partial loss of PLCZ1 function as the patient was subsequently able to conceive without AOA treatment. However, the authors postulated that the heterozygous mutations (M578T and P420L) may lead to TFF due to their presence within the catalytic domain of the protein. Collectively, these data show that many mutations have been identified in the human *PLCZ1* gene. Some of these are known to exert a more deleterious effect on protein function than others. The relative effects of these mutations appear to depend on their specific location within the *PLCZ1* gene and their specific mode of inheritance. However, although genetic screening strategies have clearly identified patients who possess *PLCZ1* mutations and that some of these mutations can impair the functionality of the PLCZ1 protein, it is highly evident that such mutations only manifest in a very small population of patients who exhibit OAD. Consequently, genetic mutations do not appear to be the main driver of PLCZ1 deficiency in the population of patients who experience fertilization failure. This hypothesis has been confirmed by our own clinical studies, carried out in collaboration with Oxford Fertility and Ninewells Hospital in Dundee (unpublished data). Despite screening over 220 patients experiencing ICSI failure, only a very small number of these patients were shown to possess PLCZ1 mutations. Of the patients screened by our diagnostic assay thus far, 37% had low levels of PLCZ1 or abnormal localization patterns, only 3% had mutations, 5% had sperm that were completely devoid of PLCZ1; however, the remainder (55%) had no PLCZ1 issues, at least using our current assays (unpublished data). However, a substantial proportion of the patients screened by our ongoing research programme were shown to exhibit clear deficiency in terms of PLCZ1 levels. These findings appear to suggest that PLCZ1 deficiency is more likely to be associated with undetermined mechanisms related to gene expression or repression, rather than mutation *per se*. At present, we do not know exactly why this is the case, although it is tempting to suspect that there are several mechanisms that can affect gene expression at the transcriptional and/or translational levels. For example, only a few studies have investigated the bidirectional promoter of *PLCZ1*; only one variant has been identified in the *CAPZA3* promoter but only in the vicinity of *PLCZ1* (Javadian-Elyaderani *et al.* 2016); the authors suspected that this variant may not affect the transcription of *PLCZ1*. Further studies are needed to fully elucidate the precise role of the bidirectional promoter with regards to the levels of gene transcription.

Indeed, multiple molecules are involved in gene expression at the transcriptional and translational levels, including RNA polymerase, enhancers, silencers, and RNA molecules, including coding and non-coding RNAs (Khalil *et al.* 2009). It would be therefore very interesting if future studies investigate whether abnormalities in these factors can impair *PLCZ1* expression, which eventually contributes to the dysfunction of the PLCZ1 protein and male infertility.

## PLCZ1 assays and the urgency to develop specific guidelines to justify the use of AOAs

Despite the beneficial effects of therapeutic approaches involving AOAs in patients that experience ICSI failure, there is an understandable and ongoing debate relating to the safety profile of these synthetic agents. Indeed, it is well-known that AOAs induce a single, prolonged Ca^2+^ transient instead of the normal oscillatory patterns. Currently, the HFEA recognizes that the use of AOAs may lead to aneuploidy during embryogenesis and has thus recommended that this treatment should be limited to infertile patients with fertilization failure and only where significant male factor has been identified (HFEA 2017). Indeed, AOA may not be the best treatment for couples with OAD (Vanden Meerschaut *et al.* 2012, Van Blerkom *et al.* 2015). Consequently, the use of AOAs should not be considered as a routine procedure in ART. Indeed, the HFEA currently considers AOAs as a treatment add-on and has placed AOA treatment on their ‘Amber’ list, thus implying that there is insufficient evidence relating to the efficiency and safety of AOAs (HFEA 2017). Only a small proportion of infertility clinics perform AOA; those that do only consider treatment according to a history of fertilization failure after ICSI or the suspicion of OAD (Tesarik *et al.* 2002, Montag *et al.* 2012, Nicopoullos *et al.* 2015). Van Blerkom *et al.* expressed significant concern over the fact that AOA treatment might become more frequently used for ICSI cycles to ensure appropriate pronuclear formation and to eliminate the potential for fertilization failure (Van Blerkom *et al.* 2015). AOA is recommended only in cases of previously failed fertilization, for couples where there is a possible defect of the sperm or the oocyte and where all other options have been exhausted. It is important that clinicians take into consideration previous cycles and patient histories before justifying the use of AOA. Furthermore, it is important that patients are aware of the procedure, and what it involves, as there are still too many unknowns associated with AOA, especially in cases where there is no specific justification. In the UK, it is a requirement to justify such treatment; however, this might not be the case in other countries where more flexibility is permitted. Van Blerkom and colleagues raised the concern that AOA could be used negligibly in ICSI procedures in non-justified cases but to eliminate the possibility of fertilization failure, thus improving success rate. AOA should only be advised to specific cases and should not be used routinely. There is a paucity of data to support the universal recommendation of AOA in clinical practice; the HFEA states that randomized, controlled trials and follow-up studies are required to specifically address the clinical use of AOAs. Given these concerns, it is imperative to establish standard guidelines for the clinical use of AOAs.

Several methods have been used to investigate patients who have experienced recurrent ICSI failure or to evaluate the ability of sperm to activate oocytes, including the heterologous injection of human sperm into mouse oocytes (MOAT) (Heindryckx *et al.* 2005), the measurement of calcium changes in human oocytes upon the injection of patient sperm (the human oocyte calcium assay) (Ferrer-Buitrago *et al.* 2018), or the assessment of centrosomal function in sperm (Terada *et al.* 2009). Electrical charges upon the sperm membrane have also been used to select sperm that contain PLCZ1; this test was considered to facilitate successful fertilization (Chan *et al.* 2006, Khakpour *et al.* 2019). However, only a limited number of clinics have introduced these approaches and a major concern is the inability of fertility units to recreate these tests as they involve specialist equipment or resources that are acquired from animal models (Heindryckx *et al.* 2005, 2008, Vanden Meerschaut *et al.* 2013). Furthermore, we know that the potency of PLCZ1 differs between the mouse and human. Therefore, it is important that essays take this disparity into consideration when developing assays that can be used in real clinical settings.

Over the years, PLCZ1 expression has been determined by measuring the levels of *PLCZ1* mRNA and RNA (Aghajanpour *et al.* 2011, Kashir *et al.* 2013, Abadi *et al.* 2016, Khakpour *et al.* 2019) or by quantifying the levels of PLCZ1 protein in sperm by immunoblotting (Yan *et al.* 2020, Yuan *et al.* 2020*b*), cell sorting (Khakpour *et al.* 2019), and immunofluorescence analysis (Kashir *et al.* 2013). In addition, other forms of dysfunction can also lead to OAD, such as DNA fragmentation in the sperm head, inadequate oocyte maturation, low oocyte numbers, or even technical problems encountered during ART. Sperm analysis remains pivotal in the evaluation of male factor infertility in clinic, and additional laboratory tests have also been proposed to patients seeking fertility treatment, including DNA integrity; thus, it is reasonable to see that researchers have attempted to correlate PLCZ1 levels, DNA fragmentation, and oxidation. However, the findings of such studies are very limited and further investigations are required before drawing firm conclusions (Taylor *et al.* 2010, Park *et al.* 2015, Tavalaee *et al.* 2017). Other studies have reported that the localization and total levels of PLCZ1 are not correlated with male age in humans although a recent study has reported otherwise in several mouse strains (Yeste *et al.* 2016*b*, Kashir *et al.* 2021). As discussed previously, it is clear that PLCZ1 is an important physiological factor that triggers oocyte activation. However, over recent years, it has become debatable whether PLCZ1 is the sole factor required for fertilization (Hachem *et al.* 2017, Nozawa *et al.* 2018, Swann 2020). Indeed, two separate studies have shown that *Plcz1*-KO mice were not sterile. Interestingly, subsequent ICSI in both studies using mutant sperm into mouse oocytes neither trigger Ca^2+^ oscillations nor oocyte activation; however, when *Plcz1* KO mice were mated with WT female mice, both obtained few, but viable, pups (Hachem *et al.* 2017, Nozawa *et al.* 2018). A recent review hypothesized that a ‘sperm cryptic-activating factor (SCAF)’ is responsible for initiating Ca^2+^ oscillations only in the absence of PLCZ1 (Jones 2018). However, what exactly SCAF is and how it activates an oocyte remain unknown. Despite conflicting debates, the overall evidence suggests that PLCZ1 is the dominant physiological trigger of normal Ca^2+^ oscillations, especially in monospermic fertilization.

It is clear that PLCZ1 has the potential to provide useful evidence for the justification of AOA in the clinical setting as required by the HFEA and in addition to basic semen analyses. Quantitative analysis of PLCZ1 immunofluorescence can provide an informative biomarker to predict the oocyte activation ability of sperm from a given patient (Kashir *et al.* 2013). Over the years, some researchers investigating PLCZ1 localization and expression in sperm have been challenged by widespread scepticism due to the polyclonal nature of the antibodies they were using and the apparent variability in the data generated by such assays. However, routine laboratory assays have successfully been developed and validated by peptide-blocking experiments, thus proving the efficacy of the protocols applied (Grasa *et al.* 2008, Heytens *et al.* 2009, Kashir *et al.* 2011*a*, 2013, Yelumalai *et al.* 2015, Yeste *et al.* 2016*b*). In a recent study, Kashir *et al.* proposed that inadequate specificity between the PLCZ1 antibody and its antigen hindered the visualization efficacy during PLCZ1 immunoanalysis and advocated the use of an antigen unmasking technique when carrying out such assays (Kashir *et al.* 2017). However, detailed experiments in our laboratory found no difference between our routine in-house assay with regards to the immunofluorescent detection of PLCZ1 when carried out with and without the antigen masking technique (Meng *et al.* 2022).

**Figure 3:**
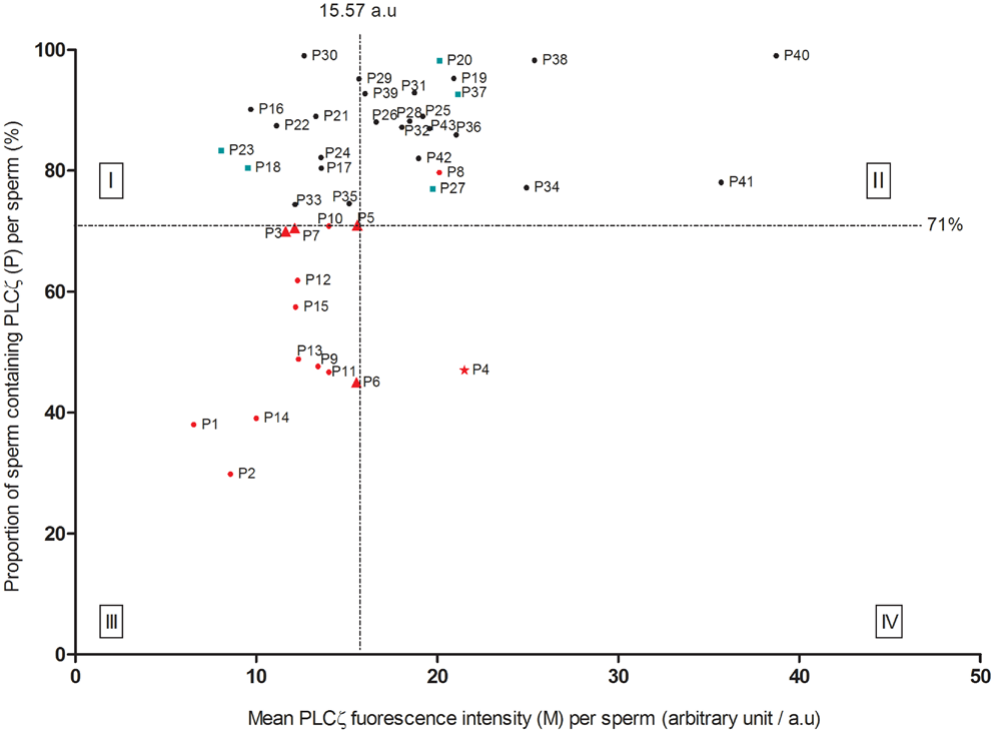
Artificial oocyte activation outcomes associated with two PLCZ1 parameters: mean PLCZ1 level in sperm (x-axis) and the proportion of sperm containing PLCZ1 (y-axis). ‘‘I, II, III, IV’ represent the four categories assigned by the two cutoffs. Patients with PLCZ1 deficiency are labelled in red (the red triangle: history with live birth with artificial oocyte activation (AOA); red dots: patients did not receive AOA; red star: patients received AOA but only chemical pregnancy). Patients who have had live births or ongoing pregnancy without AOA were labelled as green cubes, and patients with unknown outcomes of subsequent* in vitro* fertilization/intracytoplasmic sperm injection treatment were shown as black dots. For further details, see Meng *et al.* (2020). Reproduced with permission.

**Figure 4:**
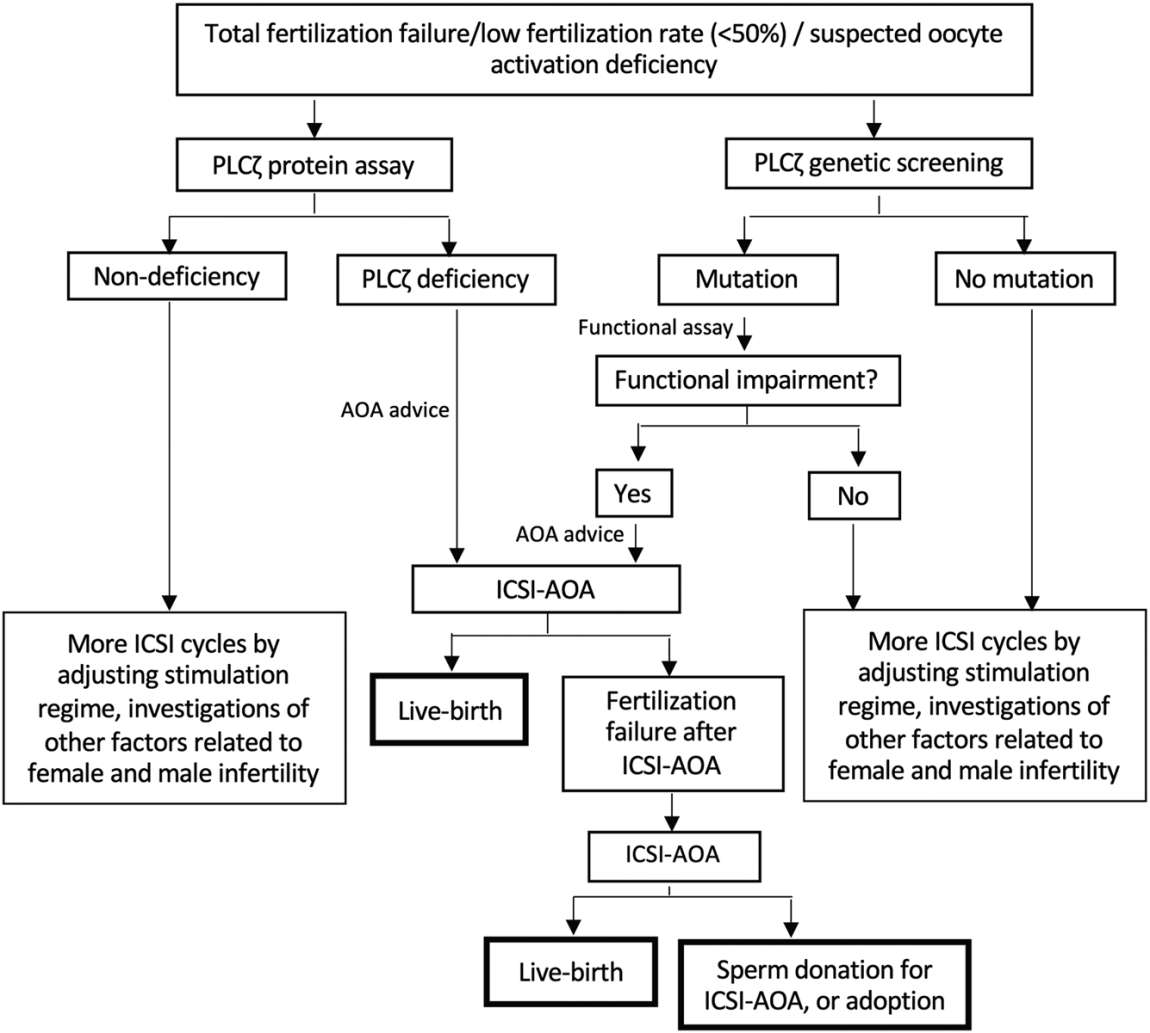
The suggested clinical algorithm for assessing the eligibility of a patient for artificial oocyte activation. AOA, artificial oocyte activation; ICSI, intracytoplasmic sperm injection. Reproduced with permission from Meng *et al.* (2020).

Immunofluorescent assays of the expression levels and localization patterns of PLCZ1 have indicated significant variation among both fertile and infertile patients. Of most concern was the fact that some infertile patients clearly exhibited lower levels of PLCZ1 than fertile controls (Meng *et al.* 2020). This indicates that the establishment of a simple ‘threshold or cut-off’ value for PLCZ1 levels for diagnostic purposes will be non-viable. Interestingly, we developed a more robust bioassay that permits the classification of ‘PLCZ1 deficiency’ by incorporating the combination of mean PLCZ1 level and the specific proportion of sperm in a given patient sample exhibiting PLCZ1 (Meng *et al.* 2020). In our study, we demonstrated that the combination of RF PLCZ1 levels and the percentage of sperm containing PLCZ1 can facilitate the selection of suitable patients for AOA treatment. In our study, we found that infertile men presenting with less than 71% of their sperm containing PLCZ1 and with a mean PLCZ1 level of 15.57 arbitrary are most likely to benefit from AOA treatment (Fig. 3). In this study, we also described the importance of using this specific threshold to diagnose patients with ‘normal’ or ‘abnormal’ sperm parameters to determine their eligibility for AOA treatment. As discussed extensively in this review, both fertile and infertile men are known to exhibit variable PLCZ1 levels; therefore, is it important that we consider both levels and the proportion of sperm containing the PLCZ1 protein before making a clinical decision. Furthermore, we found that 80% of patients exhibiting a significant ‘PLCZ1 deficiency’ (a low expression level and a low proportion of sperm exhibiting PLCZ1) who opted for AOA treatment achieved significant improvements in their fertilization rate (approximately 40% higher) and pregnancy/live birth rates (both 40% per initiated cycle), thus demonstrating that this more robust and reproducible PLCZ1 assay was more efficient with regards to diagnosing PLCZ1 deficiency (Meng *et al.* 2020). However, additional studies are now needed to help patients with a low sperm count. For example, this essay is currently limited to patients who produce a sufficient number of sperm for quantification. By using the algorithm, our laboratory-based in-house PLCZ1 assay can evaluate PLCZ1 expression in infertile males, therefore identifying whether the state of infertility is male-related (Fig. 4). This allows the development of an individually targeted strategy for the management of AOA, which can save effort, time, and cost for both IVF clinics and patients. Collectively, these findings highlight the clinical potential of PLCZ1, both as a prognostic indicator of OAD and eventually as a therapeutic protocol. While we pursue further research, it is important that we educate patients, via online forums, webinars, or awareness events, so that they fully understand the relationship between OAD and PLCZ1 and our current ability to diagnose PLCZ1 deficiency but are also aware of our current diagnostic and therapeutic limitations and the lack of evidence-based data relating to the widespread application of AOAs.

## Conclusion

Following the discovery of PLCZ1, a substantial body of evidence has confirmed the indispensable role of PLCZ1 in oocyte activation and clearly demonstrated that the expression and localization of PLCZ1 in human sperm are associated with male infertility. Infertile males show lower PLCZ1 levels in their sperm than fertile males. In addition, PLCZ1 was found to be predominantly expressed in the equatorial region of the sperm but sometimes in combination with other regions. Immunofluorescence staining is currently used to assess PLCZ1 expression and localizations in most studies, and the implementation of a robust and specific bioassay is a key requirement for future research and the evaluation of PLCZ1 levels and localization patterns to help with the justification of artificial oocyte activators in the clinic. Moreover, genetic testing of PLCZ1 can lead to the discovery of novel mutations that affect the expression/potency of the protein and therefore, oocyte activation ability. For this reason, we now need to recruit a larger cohort of fertile and infertile patients for genetic screenings so that we can gain a more comprehensive understanding of gene variations in terms of fertility status.

## Declaration of interest

The authors declare that there is no conflict of interest that could be perceived as prejudicing the impartiality of this review.

## Funding

The PLCZ1 research in our laboratory has been funded by project grants from the Royal Society (UK) and the Oxford University Medical Research Fund awarded to K Coward, the Saint Glee International Foundation (awarded to X Meng), and a European Commission, FP7-People Programme, Marie Curie-IEF (Grant Number: 626061; awarded to M Yeste), and the Nuffield Department of Women’s & Reproductive Health. Some of our PLCZ1 research has been funded by student scholarships awarded by the Governments of Malaysia and Brunei.

## Author contribution statement

C J and X M drafted the manuscript. K C revised the manuscript. All authors approved the final version for publication.
